# Repositorio de tamizaje neonatal, una herramienta para la toma de decisiones en salud pública

**DOI:** 10.7705/biomedica.7819

**Published:** 2025-09-22

**Authors:** Andrea Melissa Hidalgo, Olga Patricia Fuya, Diana Patricia Martínez

**Affiliations:** 1 Grupo Genética y Crónicas, Instituto Nacional de Salud, Bogotá, D. C., Colombia Grupo Genética y Crónicas Instituto Nacional de Salud Bogotá D. C. Colombia

**Keywords:** tamizaje neonatal, hipotiroidismo congénito, salud pública, Neonatal screening, congenital hypothyroidism, public health

## Abstract

**Introducción.:**

El tamizaje neonatal es un mecanismo esencial para detectar defectos congénitos en las primeras horas después del nacimiento.

**Objetivo.:**

Describir la capacidad técnica de los laboratorios que realizan pruebas de tamizaje neonatal en Colombia, así como la oportunidad en el reporte de la información y su cobertura en el país, a partir de los datos registrados en el repositorio de tamizaje neonatal entre enero y septiembre del 2024.

**Materiales y métodos.:**

Se analizaron 243.536 registros a nivel nacional, reportados por las instituciones prestadoras de servicios de salud que practican pruebas para tamizaje neonatal a partir de muestras de sangre seca. Se analizaron los datos correspondientes a los nacimientos ocurridos entre el 1° de enero y el 30 de septiembre del 2024. Se evaluaron los indicadores de cobertura de pruebas y la capacidad de la red diagnóstica nacional.

**Resultados.:**

La cobertura del tamizaje a nivel nacional -calculada a partir del total de laboratorios que reportaron información- fue del 71,8 %. El tiempo promedio desde el nacimiento hasta la emisión del resultado fue de 4,8 días, y solo el 62,1 % de los resultados se clasificaron como emitidos de manera "muy oportuna" (≤ 3 días). En relación con la capacidad técnica, en todo el territorio nacional, se identificaron 253 laboratorios que notifican resultados de la medición de la tirotropina *(ThyroidStimulating Hormone,* TSH).

**Conclusiones.:**

El fortalecimiento de la calidad y la oportunidad en el reporte de la información, le permitirán al país disponer de datos en tiempo real para la toma oportuna de decisiones en salud pública, que impacten positivamente la calidad de vida de los niños nacidos en el territorio colombiano.

## Abstract (en)

**Introduction.:**

Neonatal screening is an essential mechanism for the early detection of congenital anomalies within the first hours of birth.

**Objective.:**

To describe the technical capacity of the laboratories performing neonatal screening tests in Colombia, the timeliness of information reporting, and national coverage based on data from the neonatal screening repository collected during January to September of 2024.

**Materials and methods.:**

We analyzed 243,536 records nationwide uploaded by health service providers that perform neonatal screening tests on dried blood spot samples nationwide. The records corresponded to births that occurred between January 1^st^ and September 30^th^, 2024. We evaluated indicators of test coverage and capacity of the national diagnostic network.

**Results.:**

The screening coverage -calculated from the total number of laboratories that reported information- was 72.2% nationwide. The average time from birth to result release is 4.8 days, and only 62.1% of the results were classified as issued "very timely" (≤ 3 days).

**Conclusions.:**

We suggest that strengthening the quality and timeliness of information reporting will provide real-time data for timely public health decision-making, which will positively impact the quality of life of children born in Colombia.

El tamizaje neonatal constituye un mecanismo esencial para la detección temprana, el diagnóstico, el tratamiento oportuno y el seguimiento de los recién nacidos con anomalías congénitas detectables en las primeras horas o días de nacimiento, con beneficios para la salud y el desarrollo infantil ^1^. Un programa de tamizaje es una estructura integral que abarca diferentes áreas relacionadas con los recién nacidos y su entorno familiar, tales como educación, exámenes de laboratorio, manejo clínico y administración ^2^.

De acuerdo con la Resolución 207 del 2024 ^3^ y con el fin de dar cumplimiento al objeto de la Ley 1980 del 2019 ^4^, promulgada por el del tamizaje neonatal se gestione desde la estructura de un programa. Dicho programa debe tener establecidas las responsabilidades de los actores del sistema respecto al diagnóstico, el tratamiento y el inicio del seguimiento para cada una de las enfermedades detectadas mediante el tamizaje. Uno de los pilares para el buen funcionamiento del programa de tamizaje neonatal metabólico, es la disponibilidad de la información que el mismo genera, lo que le permite realizar el seguimiento de casos individuales, obtener información geográfica asociada con la cobertura del tamizaje, conocer las capacidades de los laboratorios para cubrir las necesidades del programa y determinar la cantidad de casos probables, entre otras cosas.

En respuesta a las responsabilidades delegadas por la normatividad, el Instituto Nacional de Salud desarrolló un sistema de información -el "Repositorio de tamizaje neonatal"- como una herramienta que permite el registro y el seguimiento de los infantes mediante los datos generados por los laboratorios que procesan las pruebas de tamizaje neonatal. Por lo tanto, esta plataforma representa una fuente de información en tiempo real para el análisis de los casos y la toma de decisiones, lo que favorece que el programa se fortalezca y aumente su cobertura.

El repositorio de tamizaje neonatal inició su operación en marzo del 2022, mediante la estrategia de recolección de datos denominada "incorporación de datos en tiempo real", cuyo propósito fue capturar la información directamente desde la fuente, de tal manera que la información requiera mínima o ninguna transformación.

La configuración del sistema incorpora la información consignada en la ficha que acompaña la tarjeta de la toma de muestra, la cual es diligenciada por el personal que acompaña el proceso de atención al neonato durante el parto o el egreso hospitalario.

Los bloques de información que contiene son los siguientes cuatro:

1. Información demográfica: departamento, municipio, institución responsable de la toma de la muestra, sitio de residencia de la madre y del recién nacido.
2. Información de la madre: tipo de documento y número de identificación (como fuente primaria para el rastreo de los recién nacidos), tipo de documento y número de identificación del recién nacido, nombre de
3. la madre y del recién nacido, número de hijos en el parto, empresa administradora de planes de beneficios y régimen de atención.
4. Información del recién nacido: tipo de documento y número de identificación primaria del recién nacido (certificado de nacido vivo), fecha de nacimiento y de toma de la muestra.
5. Información de la prueba: tipo de muestra, resultado de la medición según el analito, fecha de recepción de la muestra y de emisión del resultado, observaciones.

Respecto a la confidencialidad y el acceso a la información, se decidió quienes tenían acceso al repositorio según la responsabilidad de cada uno dentro del sistema de salud y el programa de tamizaje, según lo establecido en la Ley 1980 del 2019. De acuerdo con esto, fueron cinco las personas o entidades autorizadas según sus características (cuadro 1).

**Cuadro 1:** Responsabilidad y grado de acceso a la información

Perfil	Responsabilidad	Grado de acceso
Administrador	Configuración y consulta de 100 % de los datos, sin restricciones de información	Superior
Departamento	Actividades de supervisión de la cargada de los datos de los laboratorios de su red departamental	Medio: pueden consultar información de muestras tomadas en su territorio, ya sean procesadas dentro o fuera del mismo.
Empresa administradora de planes de beneficios	Actividades de supervisión del registro de datos de muestras tomadas bajo la cobertura de la empresa administradora de planes de beneficios. Cada aseguradora dispone de un usuario.	Restringido a los datos asociados con la empresa administradora de planes de beneficios que hace la consulta.
Laboratorio	Registro de datos según la ficha epidemiológica y los resultados de laboratorio	Restringido a los datos cargados por el mismo laboratorio.
Nacional	Consulta para seguimiento a casos a vigilancia epidemiológica	Medio: puede acceder a la información de las muestras cargadas en los diferentes departamentos, pero con restricción de tiempo.

Los datos son cargados en línea mediante una plantilla controlada en Excel® que, además de los datos demográficos, incluye el resultado de una o más de las siete pruebas que hacen parte del tamizaje neonatal básico para hipotiroidismo congénito, galactosemia, fenilcetonuria, fibrosis quística, hiperplasia suprarrenal congénita, déficit de biotinidasa y hemoglobinopatías.

A partir de julio del 2024, como parte de los procesos de optimización de captura de la información, se solicita el valor de la medición de los analitos evaluados y, mediante formulación, la plantilla genera la interpretación del caso como probable o descartado, según los puntos de corte establecidos a nivel nacional ^5^. Aunque a la fecha la normatividad vigente contempla un tamizaje metabólico con siete pruebas, se dispone de más información sobre hipotiroidismo congénito, ya que fue la primera enfermedad incluida en el programa nacional en el 2000 y, por esta razón, el análisis se concentra en ella.

Una vez cargados los datos, estos quedan disponibles en el servidor del Instituto, donde se consolidan y se gestionan los esquemas de almacenamiento y se origina una copia de seguridad de la información. Durante el proceso de registro y corrección de los datos, el Instituto hace un acompañamiento permanente a los usuarios (laboratorios) y a los laboratorios de salud pública para disminuir la probabilidad de errores u omitir su identificación.

## Materiales y métodos

**Figura 1:**
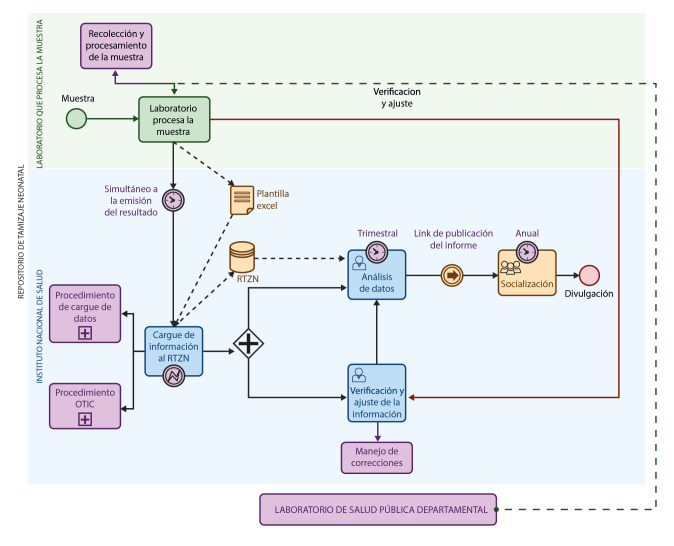
Mapa de procesos del repositorio de tamizaje neonatal

En el mapa del proceso se presenta de forma general el flujo para la recolección y la gestión de la información (figura 1). Para el presente artículo, se hizo un análisis descriptivo de la información ingresada en el "Repositorio de tamizaje neonatal", proveniente de los laboratorios de la red pública y privada que practican las pruebas establecidas por el programa en los 32 departamentos del país y el distrito capital (33 entidades territoriales). Se analizó la información de los nacidos vivos entre el 1° de enero y el 30 de septiembre del 2024.

RTZN: Repositorio del tamizaje neonatal

La información se descargó el 31 octubre del 2024 en formato Excel^®^ y, posteriormente, se revisaron los datos, considerando las siguientes categorías:

1. identificación de información duplicada, proveniente de correcciones que no fueron sustituidas oportunamente;
2. revisión de información correspondiente a nacimientos múltiples: diferenciación de registros, y
3. errores de diligenciamiento: errores de registro en la interpretación de casos.

Una vez se depuraron los datos, se procedió a su procesamiento. Se incluyó como denominador el registro de nacidos vivos disponible en la base de datos del Registro Único de Afiliados, administrada por el Ministerio de Salud y Protección Social. Se obtuvo el registro de los nacidos vivos y su lugar de nacimiento con corte del 1° de enero al 30 de septiembre del 2024.

Los datos se analizaron según los indicadores descritos en la Resolución 207 de 2024, que se aplican para la supervisión de la implementación del tamizaje endocrino metabólico, y cuya cobertura se presenta por departamento y por regiones, como sigue:

- Cobertura de tamizaje de hipotiroidismo congénito: se deben evaluar muestras y nacidos vivos en el mismo periodo.
- Oportunidad del tamizaje para hipotiroidismo congénito: días transcurridos desde el nacimiento hasta la emisión del resultado.
- Proporción de niñas versus niños con probable hipotiroidismo congénito según la tirotropina *(Thyroid Stimulating Hormone,* TSH): definida como el número de niñas respecto al de niños con un valor de la TSH indicativo de un caso probable de hipotiroidismo congénito.
- Capacidad técnica: número de laboratorios que hacen tamizaje neonatal en el país y su distribución por entidades territoriales.

## Resultados

Con la información recopilada en el repositorio del 1^o^ de enero al 30 de septiembre del 2024, se analizaron la cobertura y la oportunidad del tamizaje en el país, la proporción de niños versus niñas tamizados y la capacidad técnica de los laboratorios que practican pruebas de tamizaje.

En la fase de revisión, se excluyeron nueve registros duplicados y 192 de partos gemelares que no estaban plenamente identificados, según la guía para el registro denominada "catálogo de variables". Se identificaron errores de registro, por lo que se solicitó a los laboratorios la corrección de la información según el proceso establecido por el Instituto.

### Cobertura de tamizaje

**Figura 2:**
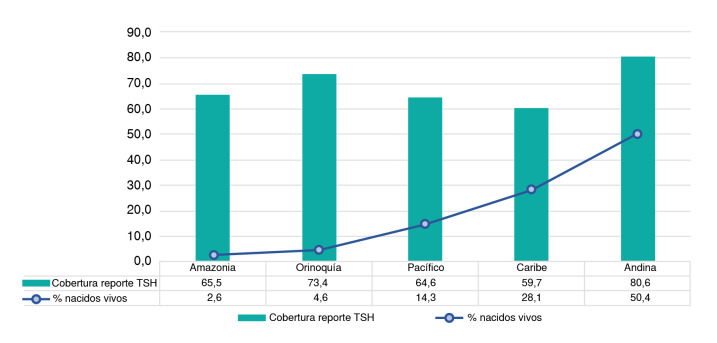
Cobertura de tamizaje para hipotiroidismo congénito respecto al número de nacimientos por región

Como se observa en la figura 2, de los 330.233 nacidos vivos, se pudo analizar la información de 330.225, ya que ocho no disponían de información confirmada del lugar de nacimiento. El 78,5 % de los nacimientos se concentraron en las regiones Andina (50,4 %) y Caribe (28,1 %).

La región Andina presentó la cobertura de tamización de hipotiroidismo congénito más alta (80,6 %), seguida por la registrada en la región de la Orinoquía (73,4 %), la Amazonia (65,5 %), el Pacífico (64,6 %) y el Caribe (59,7 %). A nivel nacional, se registra una cobertura del 71,8 % (236.985 muestras reportadas de 330.233 nacidos vivos).

TSH *(ThyroidStimulating Hormone):* tirotropina

**Figura 3:**
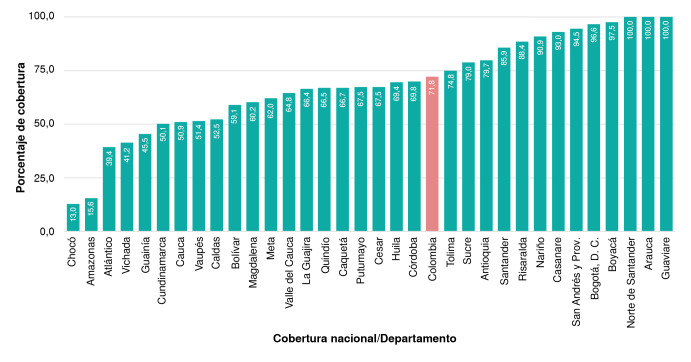
Porcentaje de cobertura de la toma de muestras para el tamizaje de hipotiroidismo congénito por entidad territorial. Se reporta también el porcentaje de cobertura nacional.

Trece departamentos registran coberturas superiores a la media nacional (71,8 %) y, en siete de ellos, la cobertura es superior al 90 % (Casanare, Arauca, Archipiélago de San Andrés y Providencia, Nariño, Norte de Santander, Boyacá, y Guaviare). Aunque en algunos departamentos el análisis de la mayoría de las muestras ocurre fuera del territorio, esto no influenció el indicador. Se destaca que los departamentos de Guaviare, Arauca y Norte de Santander tienen registrada la información del 100 % de los niños nacidos en sus territorios, sin embargo, podrían presentarse errores de registro que sobredimensionen la cobertura (figura 3). Cabe resaltar que el 83,3 % de los departamentos de la región amazónica muestran coberturas inferiores a la media nacional (71,8 %), mientras que, para los de la región Caribe y Pacífica, se estimó una cobertura inferior al 75 % para hipotiroidismo congénito.

### Oportunidad del tamizaje

Con los datos registrados en el repositorio de tamizaje neonatal, fue posible medir indicadores de oportunidad para cada una de las etapas cruciales al evaluar la eficacia de un programa de tamizaje neonatal. Como estrategia de supervisión general del programa a nivel nacional, se proponen tres indicadores de oportunidad relacionados con el tamizaje de hipotiroidismo congénito y las categorías para su medición (cuadro 2), basadas en los indicadores establecidos en el programa de tamizaje mexicano ^6^.

**Cuadro 2:** Componentes de la oportunidad del tamizaje de hipotiroidismo congénito

Criterio	Medición	Descripción	Categorías
Oportunidad del tamizaje	Días transcurridos desde el nacimiento hasta la emisión del resultado	Aportar evidencia para tener un panorama general sobre el tiempo promedio que tarda la ejecución del tamizaje	Muy oportuno: ≤ 3 días Oportuno: ≤ 7 días No oportuno: > 7 días
Oportunidad de remisión o transporte de muestras	Días trascurridos desde la toma de la muestra hasta la recepción por el laboratorio que la procesa	Aportar información sobre la eficiencia del programa de tamizaje en relación con la logística de la movilización de las muestras	Muy oportuno: ≤ 0 días Oportuno: ≤ 3 días No oportuno: > 3 días
Oportunidad de acceso a la información	Días trascurridos desde la emisión del resultado hasta su notificación en el repositorio	Aportar información para determinar la observancia de los laboratorios a la notificación establecida en la Resolución 207 del 2024.	Muy oportuno: ≤ 1 días Oportuno: ≤ 3 días No oportuno: > 3 días

Para la evaluación de las entidades territoriales, se midió el porcentaje de cumplimiento en la categoría de "muy oportuno". Aquellas que alcanzaron un porcentaje mayor o igual al 80 %, se clasificaron como "muy oportunos", aquellos entre el 60 % y 79,9 %, como "oportunos", y con menos del 60 %, como "no oportunos".

En el cuadro 3 se observa la clasificación general por departamentos según cada criterio de oportunidad del tamizaje. Se destaca que los de Bolívar, Cesar, Huila, Nariño, Norte de Santander, Quindío, Risaralda y Sucre tienen en general un tamizaje "muy oportuno" (evaluado desde la toma de la muestra hasta la emisión del resultado). Por su parte, Amazonas, Atlántico, Casanare, La Guajira, Magdalena y Santander hacen un tamizaje "oportuno". Esto resultados indican que el 48,5 % de las entidades territoriales tiene un tiempo adecuado para el tamizaje.

**Figura 4:**
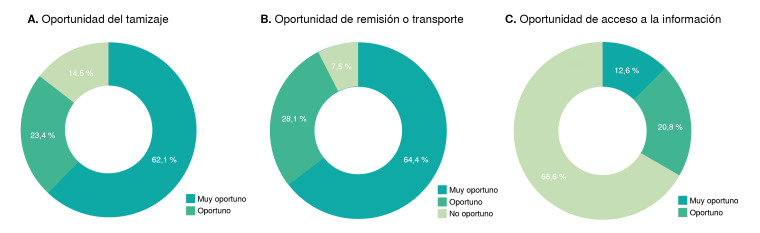
Tiempos de ejecución del tamizaje neonatal en Colombia

Una vez identificado el comportamiento de cada departamento y del Distrito Capital, se evaluó cada aspecto de la oportunidad del tamizaje a nivel nacional (figura 4). En la figura 4A se observa que se requieren 4,8 días en promedio, para acceder a un resultado del tamizaje de hipotiroidismo congénito. El 62,1 % de los registros notificados evidenciaron que el acceso a la tamización se hace posible dentro de los primeros tres días después del nacimiento.

**Cuadro 3:** Resultados por criterio de los componentes de la oportunidad del tamizaje

Entidad territorial	Oportunidad		
	Tamizaje	Remisión o transporte	Acceso a la información
Amazonas	Oportuno	Muy oportuno	No oportuno
Antioquia	No oportuno	Oportuno	No oportuno
Arauca	No oportuno	No oportuno	No oportuno
Atlántico	Oportuno	Oportuno	No oportuno
Bogotá	No oportuno	No oportuno	No oportuno
Bolívar	Muy oportuno	Muy oportuno	No oportuno
Boyacá	Oportuno	Muy oportuno	No oportuno
Caldas	No oportuno	No oportuno	No oportuno
Caquetá	No oportuno	Muy oportuno	No oportuno
Casanare	Oportuno	Muy oportuno	No oportuno
Cauca	No oportuno	No oportuno	No oportuno
Cesar	Muy oportuno	Muy oportuno	No oportuno
Chocó	No oportuno	Muy oportuno	No oportuno
Córdoba	No oportuno	No oportuno	No oportuno
Cundinamarca	No oportuno	No oportuno	No oportuno
Guainía	No oportuno	No oportuno	No oportuno
Guaviare	No oportuno	No oportuno	No oportuno
La Guajira	Oportuno	Muy oportuno	No oportuno
Huila	Muy oportuno	Muy oportuno	No oportuno
Magdalena	Oportuno	Oportuno	No oportuno
Meta	No oportuno	No oportuno	No oportuno
Nariño	Muy oportuno	Muy oportuno	No oportuno
Norte de Santander	Muy oportuno	Oportuno	No oportuno
Putumayo	No oportuno	Oportuno	No oportuno
Quindío	Muy oportuno	No oportuno	No oportuno
Risaralda	Muy oportuno	No oportuno	No oportuno
San Andrés y Providencia	No oportuno	Muy oportuno	No oportuno
Santander	Oportuno	Oportuno	No oportuno
Sucre	Muy oportuno	Muy oportuno	No oportuno
Tolima	No oportuno	No oportuno	No oportuno
Valle del cauca	No oportuno	No oportuno	No oportuno
Vaupés	No oportuno	No oportuno	No oportuno
Vichada	No oportuno	Oportuno	No oportuno

Oportunidad de tamizaje: días desde el nacimiento hasta la emisión del resultado

Oportunidad de remisión o transporte: días desde la toma de la muestra hasta su recepción en el laboratorio que la procesa

Oportunidad de acceso a la información: días trascurridos desde la emisión del resultado hasta su notificación en el repositorio

En la figura 4B, se observa que el transporte de la muestra al laboratorio toma aproximadamente 1,1 días, es decir, el 64,4 % de las muestras se recibieron de manera "muy oportuna" en los laboratorios de la red de tamizaje neonatal. Respecto a la oportunidad de la notificación de los resultados al repositorio de tamizaje neonatal (figura 4C), el promedio nacional es de 15 días. Esto significa que el 66,6 % de los reportes se notifica de forma "no oportuna", ya que la legislación establece que debe hacerse simultáneamente con la emisión del resultado del tamizaje, lo que facilitaría la implementación de intervenciones adecuadas que modifiquen el curso de la enfermedad y, por lo tanto, el pronóstico del recién nacido.

### Proporción entre niñas y niños tamizados

A partir del Registro Único de Afiliados se obtuvo el registro de 330.225 nacidos vivos, de los cuales 160.819 (48,7 %) fueron de sexo femenino, 169.388 (51,3 %) de sexo masculino y en 18 (0,01 %) no se reportó el sexo.

Según los indicadores establecidos en la resolución, los casos probables de hipotiroidismo congénito se distribuyen en una proporción 1:1,3 (por cada niña identificada como caso probable para hipotiroidismo congénito hay 1,3 niños). En el cuadro 4 se muestra la proporción para cada departamento.

### Capacidad técnica de la red de laboratorios

En todo el territorio nacional, se identificaron 253 laboratorios que notifican resultados de la TSH. No se encontraron laboratorios que procesen muestras para tamizaje neonatal en el departamento de Guaviare, ya que las muestras son remitidas a tres laboratorios ubicados en la región Andina.

**Figura 5:**
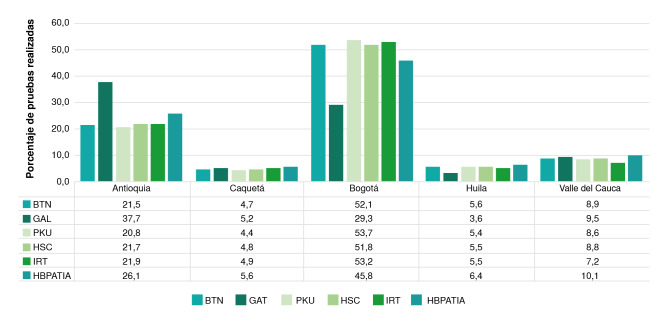
Ubicación por departamento de los laboratorios que hacen pruebas diferentes a la medición de la TSH. Se muestra el porcentaje de muestras procesadas

Respecto a los laboratorios que ofrecen la medición de analitos diferentes a la TSH, la oferta de pruebas está concentrada en cuatro departamentos y en el distrito capital (Bogotá), y en 13 laboratorios, lo que corresponde al 5,1 % de los inscritos en el repositorio de tamizaje neonatal durante el periodo analizado (figura 5). De estas cinco entidades territoriales, se encontró que en Bogotá se procesan más del 50 % de las pruebas de todo el país, seguida de Antioquia (22 %). En este último departamento, se practica el 37 % de las pruebas para tamizaje de galactosemia.

**Figura 6:**
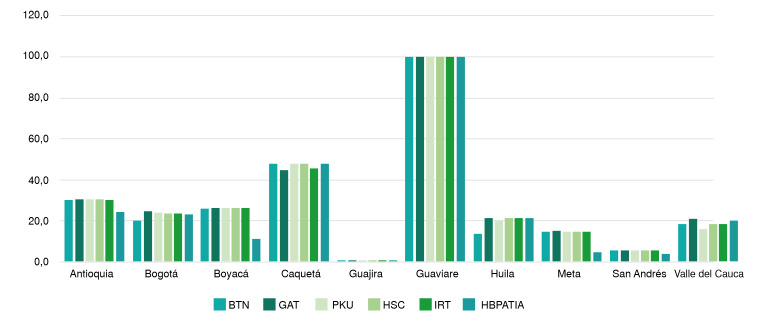
Porcentaje de muestras reportadas por cada departamento que ha implementado pruebas diferentes a la de la tirosina (TSH) en el tamizaje neonatal

BTN: biotinidasa; GAL: galactosemia; PKU: fenilcetonuria; HSC: hiperplasia suprarrenal congénita; IRT: tripsinógeno inmunorreactivo (fibrosis quística); HBPATIA: hemoglobinopatías

Dando alcance a la cobertura de pruebas diferentes a la determinación de la TSH y como parte de la progresividad planteada por el programa de tamizaje neonatal, en el país se practican pruebas para evaluar galactosemia, fenilcetonuria, fibrosis quística, hiperplasia suprarrenal congénita, déficit de biotinidasa y hemoglobinopatías. Como se observa en la figura 6, se registraron alrededor de 32.000 nacidos vivos a quienes se les efectuó el tamizaje para estas enfermedades, lo que significa que solo entre el 9 y el 11 % de los nacidos vivos notificados al repositorio lograron acceder al tamizaje neonatal básico completo o, al menos, a una de las pruebas diferentes a la de la TSH.

**Cuadro 4:** Proporción probable entre niñas y niños con tamizaje de hipotiroidismo congénito

Departamento	Proporción
Amazonas	1:0
Antioquia	1:1,3
Arauca	1:2,2
Atlántico	1:1,3
Bogotá	1:1,4
Bolívar	1:2
Boyacá	1:1
Caldas	1:2,4
Caquetá	1:0,7
Casan are	1:0,3
Cauca	1:1
Cesar	1:1,1
Chocó	1:1
Córdoba	1:1,3
Cundinamarca	1:1,7
Guaviare	1:1,3
Huila	1:1,2
La Guajira	1:1,3
Magdalena	1:1
Meta	1:2,8
Nariño	1:1,8
Norte de Santander	1:1
Putumayo	1:2,4
Quindío	1:1,5
Risaralda	1:1,5
San Andrés y Providencia	1:1
Santander	1:1,3
Sucre	1:1,3
Tolima	1:0,8
Valle del cauca	1:1,3
Vaupés	1:3

BTN: biotinidasa; GAL: galactosemia; PKU: fenilcetonuria; HSC: hiperplasia suprarrenal congénita; IRT: tripsinógeno inmunorreactivo (fibrosis quística); HBPATIA: hemoglobinopatías

En departamentos como el del Guaviare, la totalidad de las muestras son sometidas a las seis pruebas adicionales incluidas en el programa. Se encontró que ocho departamentos más aplican estas pruebas adicionales a la medición de la TSH: Atlántico, Bolívar, Córdoba, Cundinamarca, Norte de Santander, Quindío, Risaralda y Tolima. Sin embargo, estas no representan más del 1 % de las muestras analizadas, por lo que no se incluyeron en la gráfica.

Al analizar los datos fue posible determinar que existe una estrecha relación entre la capacidad técnica y el uso que se puede hacer de ella, y esto tiene que ver con el origen de la muestra empleada para el tamizaje neonatal. Se observó que el 83,9 % de las muestras con las que se realizan las pruebas provienen de sangre de cordón umbilical, mientras que el 16,0% proviene de sangre seca de talón, la cual es más difícil de procesar.

## Discusión

La capacidad técnica del país para realizar el tamizaje neonatal y su relación con la progresividad en su implementación, presentan dos escenarios: el primero tiene que ver con la oferta de los laboratorios según las técnicas disponibles para el procesamiento de las muestras; se destaca a aquellos laboratorios que ofrecen pruebas diferentes a la medición de la TSH. El porcentaje de estos laboratorios (5,1 %) se concentra en cuatro departamentos y el distrito capital, lo que plantea la necesidad de decidir cuál sería la estrategia más adecuada para garantizar el acceso al tamizaje. En otros países de Latinoamérica, se ha propuesto la centralización o la regionalización del programa de tamizaje, con el fin de disminuir los tiempos de respuesta, optimizar la movilización de las muestras, mejorar los costos del programa, asegurar la cobertura y revisar la eficacia de los puntos de corte asignados ^7^.

El segundo escenario tiene que ver con el origen de las muestras. En Colombia, se encuentra implementada la toma de muestra de sangre de cordón umbilical. No obstante, en concordancia con la legislación vigente y para hacer efectiva la progresividad, es necesaria la transición a la toma de muestra sanguínea del talón, que a la fecha alcanza solo el 16 % de las muestras tomadas. La transición implica considerar la logística para la toma de muestra del recién nacido entre las 48 y las 72 horas de vida (intervalo requerido para evaluar adecuadamente la mayoría de los analitos) y la capacitación del personal en la técnica bajo los criterios internacionales descritos en la norma NBS01-A6 del *Clinical and Laboratory Standards Institute* (CLSI) ^8^.

Respecto a la cobertura nacional, se evidencia que los territorios con barreras de orden público, difícil acceso geográfico o escasos o ningún laboratorio (por ejemplo, Guaviare, Arauca o Nariño), en los cuales se esperaría una baja cobertura, son ejemplo de una implementación exitosa del programa. Los resultados muestran que, aun con variables como el transporte de muestras a otros territorios, la cobertura del tamizaje se mantiene, lo que indica una articulación adecuada de todos los actores involucrados en el programa. Un panorama diferente se presenta en aquellos departamentos cuya cobertura es inferior a la media nacional. Aunque algunos de ellos tienen mejores condiciones sociodemográficas y económicas, estas no se reflejan en la cobertura y la oportunidad del tamizaje. Esto requiere acciones inmediatas -lideradas desde el nivel territorial- para fortalecer la articulación del programa mediante actividades encaminadas al desarrollo de competencias del personal de salud, mejoras en la logística para la remisión y el transporte de muestras, entre otros.

La cobertura nacional alcanzada durante el periodo evaluado (71,8 %) dista de la planteada en revisiones anteriores, que señalan para Colombia coberturas del 80 al 81 % 7,9. Sin embargo, se observó un incremento importante en comparación con la cobertura alcanzada en el 2023, que fue del 56,9 % ^9^. La región Andina concentra el mayor número de nacimientos, pero preocupa que la región Caribe -que aporta el 28 % de los nacimientos-presente una cobertura deficiente (59,7 %). Por su parte, las regiones del Pacífico (64,6 %), de la Orinoquía (73,4 %) y de la Amazonía (65,5 %), aunque tampoco alcanzaron el porcentaje de cobertura esperado, este sí aumentó respecto al 2023, año en el que alcanzaron coberturas del 58,3, 69,2 y 53,8 %, respectivamente ^10^.

Es importante destacar que el reporte al repositorio de tamizaje neonatal está condicionado por la oportunidad en la notificación que, según lo establecido en la Resolución 207 del 2024, debe ser inmediata después de la emisión de los resultados. A pesar de esto, el sistema de información les permite a los laboratorios cargar registros con 30 días de retraso respecto a la fecha de toma de la muestra.

A pesar de lo anterior, se han presentado casos de laboratorios que no lo hacen dentro de este intervalo de tiempo, por lo que dichos datos quedan excluidos del sistema. Algunos laboratorios han manifestado que esto se debe a problemas en la disponibilidad de talento humano, acceso limitado o fallido a internet y rotación de personal, entre otros. Para ello, se han creado estrategias de acompañamiento gestionadas por los laboratorios de salud pública, como asistencias técnicas, la aplicación de estándares de calidad según la Resolución 1619 del 2015 y actividades de seguimiento que ayudan a normalizar la implementación de la notificación según la Resolución 207.

Se han detectado casos en los que las muestras se remiten hasta dos meses después de su toma. Esto hace necesario identificar los factores determinantes que afectan la oportunidad, desde la misma gestión de las empresas administradoras de planes de beneficios hasta las instituciones prestadoras de servicios de salud que atienden partos. A partir de ello, se deben implementar acciones eficaces que permitan el manejo oportuno de las muestras y los casos, con su respectivo seguimiento.

El porcentaje de oportunidad del tamizaje -desde el nacimiento hasta la emisión del resultado- apenas alcanzó el 48,5 %, situación que afecta el diagnóstico y el tratamiento oportuno de los casos de hipotiroidismo congénito. Las razones que condicionan esta situación en los departamentos con baja oportunidad, pueden estar relacionadas con la subcontratación de la prestación del servicio fuera de los territorios, problemas en la logística del transporte en lugares con barreras geográficas o la escasa cantidad de muestras que lleva a las empresas administradoras de planes de beneficios a hacer una recolección acumulada para mejorar los costos, lo que impacta la calidad analítica de las mediciones y la efectividad del programa.

La oportunidad de notificación al repositorio de tamizaje neonatal se debe reforzar desde la autoridad local, con la divulgación de la normatividad y el seguimiento a los laboratorios. Esta falta de oportunidad se relaciona con dificultades de los laboratorios para coordinar los procesos de notificación, por lo que conduce a un cargue acumulado de datos, con frecuencia semanal o quincenal.

El alcance de los grados de cobertura esperada (> 80 %) en todo el territorio nacional -como indicador mínimo de implementación y notificación-y la mejora en la oportunidad (que cada departamento alcance el 80 % en cada aspecto y se ubique en la categoría "muy oportuno"), se traducirían en un mayor impacto del tamizaje neonatal. Asimismo, se debe garantizar que el país haga la transición de la toma de muestra de sangre de cordón umbilical a la de talón, junto con la implementación del tamizaje metabólico básico, conforme a las recomendaciones del Instituto Nacional de Salud y la normatividad vigente ^5^. El porcentaje actual (16 %) de muestras de sangre de talón (16 %) requiere de acciones inmediatas o a muy corto plazo para garantizar el éxito en la progresividad del programa de tamizaje metabólico.

El análisis de la información recolectada en el repositorio de tamizaje neonatal permite ver el crecimiento que ha tenido el programa, ya que pasó de un registro de 90 laboratorios en el 2022 a 253 en septiembre del 2024. Entre los factores clave se encuentran el ajuste normativo de la Resolución 207 de 2024, las mejoras propias del sistema de carga de datos al repositorio, las asistencias técnicas y las actividades de seguimiento que implementó el Instituto durante el 2023 y el 2024, elementos que en conjunto han contribuido al fortalecimiento y crecimiento del repositorio.

La información presentada permite conocer la situación actual del tamizaje en Colombia, así como reconocer las áreas que requieren atención inmediata. Por esta razón, se puede afirmar que el repositorio de tamizaje neonatal es una excelente herramienta para consolidar información que permita una toma de decisiones objetiva con impacto positivo en las acciones de salud pública y que posicione al tamizaje neonatal como un programa eficaz.

Otras herramientas diseñadas por el Instituto, como los programas de evaluación de desempeño y el acompañamiento a los laboratorios de salud pública para fortalecer su competencia técnica, complementan el programa de tamizaje neonatal y ayudan a que este alcance el grado de los referentes en Latinoamérica. Con un programa de tamizaje neonatal eficaz, se busca impactar la calidad de vida de los niños que nacen en el territorio colombiano mediante la detección oportuna de las enfermedades incluidas, lo que contribuye a disminuir las discapacidades, y mejorar el bienestar de los niños y sus familias.

A partir de los resultados presentados y su discusión, se puede concluir que el repositorio de tamizaje neonatal es una herramienta que aporta información objetiva para evaluar de forma sistemática la implementación del programa de tamizaje neonatal metabólico en Colombia. Se sugiere que, a partir de los resultados presentados, se orienten estrategias específicas en cada territorio para aumentar la cobertura del programa, de manera que los datos sean suficientes para la adecuada operación del programa de tamizaje neonatal.
